# Mechanisms of Intracranial Aneurysm Rupture: An Integrative Review of Experimental and Clinical Evidence

**DOI:** 10.3390/jcm14228256

**Published:** 2025-11-20

**Authors:** Masahiko Itani, Tomohiro Aoki

**Affiliations:** 1 Department of Neurosurgery, Kyoto University Graduate School of Medicine, Kyoto 606-8507, Japan; masa9426@kuhp.kyoto-u.ac.jp; 2 Department of Pharmacology, The Jikei University School of Medicine, Tokyo 105-8461, Japan

**Keywords:** intracranial aneurysm, rupture mechanism, hemodynamics, inflammation, genetics, vessel-wall MRI

## Abstract

**Background**: Intracranial aneurysm (IA) rupture is a devastating event in neurosurgery and a leading cause of subarachnoid hemorrhage. Although aneurysm size has been traditionally emphasized, recent research has highlighted multifactorial mechanisms involving hemodynamic stress, wall degeneration, inflammation, and genetic predisposition. **Methods**: Evidence from animal models, human pathological studies, computational fluid dynamics analyses, genetic association studies, and advanced imaging research was reviewed to provide an integrated view of rupture mechanisms. **Results**: Morphological and hemodynamic studies have shown that high aspect and size ratios, coupled with low wall shear stress and an elevated oscillatory shear index, contribute to focal wall weakening. Histopathological analyses of ruptured aneurysms consistently reveal endothelial loss, smooth-muscle-cell depletion, extracellular matrix degradation, and intense inflammatory cell infiltration, with patterns such as extremely thin, hypocellular, thrombosis-lined walls. Experimental studies have identified active inflammatory pathways, including neutrophil-driven cascades via CXCL1 signaling and complement C5a–C5aR1 activation, as direct triggers of wall failure. High-resolution vessel-wall magnetic resonance imaging correlates contrast enhancement with histological evidence of inflammation and neovascularization, suggesting its utility as a biomarker of instability. **Conclusions**: IA rupture is driven by a dynamic interplay between adverse hemodynamic environments, inflammatory degeneration, genetic susceptibility, and pathological vascular remodeling. Integrating these mechanistic insights may improve risk stratification and guide the development of targeted preventive strategies.

## 1. Introduction

The most serious problem associated with intracranial aneurysms (IAs) is subarachnoid hemorrhage (SAH) due to rupture. Given that outcomes after SAH remain poor despite advances in treatment, systemic management, and critical care, strategies for preventing aneurysm rupture are essential.

In recent years, the improved accuracy and accessibility of diagnostic modalities, such as magnetic resonance imaging (MRI) and the widespread use of routine health examinations, have led to an increased detection rate for unruptured intracranial aneurysms. Preventive medical strategies have become feasible as unruptured aneurysms are detected earlier; however, despite risk factor control (e.g., smoking and blood pressure management), options remain largely limited to surgical or endovascular interventions. This demonstrates the need to reveal the mechanisms underlying IA rupture to enable reliable risk prediction and determine appropriate treatment indications. Moreover, as no effective pharmacological or noninvasive therapies are currently available, the development of novel treatments targeting rupture-inducing mechanisms is of significant clinical and societal importance.

Methods: A narrative review of experimental, pathological, imaging, and clinical cohort studies published in English and Japanese was conducted. The search sources included PubMed searches, reference lists of key reviews, and prospective cohort studies. Studies that (i) prospectively recorded rupture or growth outcomes, (ii) linked imaging biomarkers to histological or clinical events, and (iii) provided quantitative or externally validated tools were prioritized. Reference numbering followed the existing list, and additional clinical studies were added where necessary to complement cohort-level determinants, analysis of post-growth risk, and imaging biomarkers.

## 2. Knowledge of Mechanisms That Lead to Rupture

### 2.1. Overview of Mechanisms of Aneurysm Initiation and Growth

**Figure 1:**
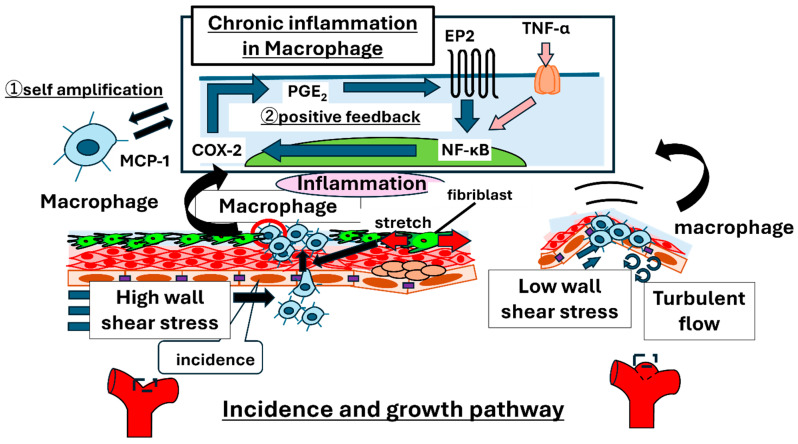
Schematic illustration of the formation and growth of intracranial aneurysms. Hemodynamic forces, including shear stress and wall tension derived from upstream blood flow, activate macrophages, leading to the initiation and progressive enlargement of aneurysms.

Before explaining rupture, the current knowledge on the incidence and growth of IAs is summarized (Figure 1). Clinical materials and animal models have provided evidence that allows for the gain or loss-of-function of candidate factors controlling pathobiology, thereby clarifying the causal links between specific factors and diseases. These studies have delineated the mechanisms that contribute to the incidence and growth of IAs [1,2,3,4,5].

IA formation at arterial bifurcations is regulated by hemodynamic loading, specifically elevated shear stress and stretch. High shear stress activates endothelial cells, inducing adhesion molecules and chemokines such as MCP-1 (monocyte chemoattractant protein-1), thereby recruiting macrophages. Stretching activates adventitial fibroblasts to produce macrophage chemoattractants [6,7]. These two distinct forces cooperate to recruit macrophages to the cell wall, thereby triggering inflammation [6,8,9,10,11,12]. Consequently, the IA sites may be defined by these two hemodynamic loadings [8,13,14]. Furthermore, endothelial activation by high shear stress promotes the dedifferentiation and migration of smooth muscle cells, inducing intimal thickening akin to atherosclerotic lesions, which, together with macrophages, becomes a focal source of inflammation [15].

Inside an established IA wall, a low shear stress prevails [8,16]. Image-based computational fluid dynamics (CFD) in the models further indicated that the growth regions were characterized by low shear stress and turbulent flow. Low shear stress alone enhances macrophage infiltration, and turbulent flow and low-shear stress work together to induce endothelial expression of macrophage adhesion/trafficking factors [16,17], thus maintaining macrophage infiltration throughout growth, although the load state differs from that in the incidence phase.

Accordingly, macrophage-dependent inflammation occurs from the incidence to the growth stage [16]. Normally, transient inflammation is sustained by mechanisms such as the upregulation of receptors for pro-inflammatory mediators, cooperative pathway crosstalk, and positive feedback loops [18]. Ultimately, proteases (e.g., matrix metalloproteinase-2,9 (MMP-2,9)) from macrophages and activated vascular cells degrade the extracellular matrix, while reactive oxygen species (ROS) and cytokines cause tissue damage, weakening the cell wall and enabling initiation and growth.

### 2.2. Recent Insights into Mechanisms of IA Rupture

**Figure 2:**
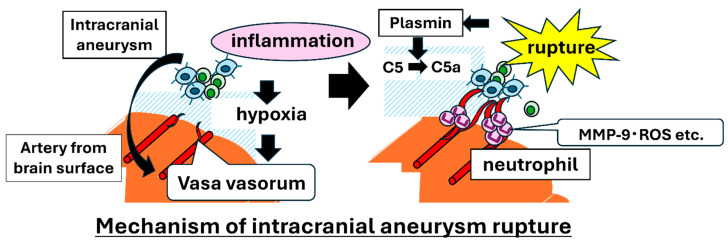
Schematic illustration of intracranial aneurysm rupture. A hypoxic microenvironment around the aneurysm induces neovascularization from the brain parenchyma and promotes neutrophil infiltration into the aneurysmal wall.

The central question is whether rupture represents a continuum of growth or a qualitatively distinct process. Clinically, many unruptured IAs remain stable for years with an annual rupture risk of approximately 1% [19]. Yet some long-stable aneurysms suddenly change in morphology and rupture [20,21], while others rupture soon after initial detection, including in younger patients. These observations suggest that rupture requires mechanisms that differ from those involved in growth. Recent studies support this hypothesis (Figure 2) [22,23,24,25].

Macrophages and neutrophils are present at ruptured points [26,27,28,29], with changes in macrophage subsets [30,31]. Whether inflammation is causal or secondary to rupture remains unclear, given that SAH itself provokes inflammatory responses via intracranial hypertension and clot formation [28,32]. The establishment of animal models of spontaneous rupture has highlighted the importance of inflammation during rupture [22,23,24,25,33,34].

A key finding was the presence of adventitial neovessels (vasa vasorum) in rupture-prone lesions. However, normally, there is no vasa vasorum in the arteries of the brain. In animals and humans, neovessels are often found near rupture points, implicating angiogenesis in rupture induction, and vascular endothelial growth factor is a candidate mediator. The neovessels provide a new route for leukocyte entry beyond the luminal endothelium. Around these vessels, many macrophages accumulate, and distinct from the growth phase, neutrophil accumulation is prominent. Single-cell transcriptomics can also detect cell populations that are consistent with neutrophils in IA tissues. Experimentally, increasing the number and activation of neutrophils (e.g., G-CSF (granulocyte colony-stimulating factor)) promotes rupture, establishing a functional role for neutrophils [24,25,35].

Mechanistically, neovessel-recruited neutrophils produce MMP-9, a collagen-degrading enzyme that drives the destruction of the extracellular matrix [23]. Given the strong capacity for ROS generation and the release of cytokines, severe tissue injury is likely to occur under an intensified inflammatory state [23]. Neutrophil-dependent inflammation may also activate and induce macrophage infiltration, thereby amplifying local inflammation. Therefore, during rupture, the chronic inflammatory niche undergoes both structural (angiogenesis) and qualitative (involvement of neutrophils) transformations [36].

A potential mediator of neutrophil recruitment, C5a, has been identified [37]. Intriguingly, although C5a was detected in IA lesions, the classical upstream complement components (C3a and membrane attack complex) were not. Instead, locally generated plasmin, produced via tissue-type plasminogen activator (t-PA)-driven fibrinolysis, can cleave complement component 5 (C5) into C5a, suggesting that coagulation/fibrinolysis activation generates C5a in situ to attract neutrophils [37,38]. The origin of neovessel-driven rupture has been examined via tissue clearing and 3D imaging; neovessels arise from cortical surface vessels and extend toward rupture points [25]. Because intracranial arteries lack a nutritive vasa vasorum and are in the subarachnoid space, their adventitia is physiologically hypoxic; inflammation increases cellularity and activation, worsening hypoxia, which then induces angiogenesis in the aneurysm wall [25]. Altogether, the rupture process appears to be governed by hypoxia and aggravated by macrophage-dependent chronic inflammation, which induces angiogenesis and establishes a neutrophil-inclusive inflammatory microenvironment, culminating in MMP-9–mediated (and related) severe tissue destruction [25,39].

## 3. Clinical Implications of Defining IAs as a Chronic Inflammatory Disease

Acknowledging IAs as a disease of hemodynamic stress–induced chronic inflammation and its structural/qualitative transitions indicates clear translational avenues: the development of anti-inflammatory pharmacotherapies, risk prediction metrics targeting adverse hemodynamics, and flow-modifying strategies [40,41,42].

In animal models, multiple drugs targeting inflammatory mediators have been shown to suppress initiation, growth, and rupture, implying the feasibility of medical therapy in humans. Observational data also suggest that statins, which are anti-inflammatory lipid-lowering drugs, may reduce the risk of SAH due to aneurysm rupture [18,31,43]. In the diagnostic fields, macrophage imaging using ferumoxytol-enhanced MRI shows promise for risk stratification. Moreover, CFD based on computed tomography (CT)/MRI geometry and increasingly Artificial Intelligence (AI)-augmented imaging aim to deliver immediate, patient-specific rupture risk following imaging [44,45,46]. Thus, mechanistic advances in rupture research are paving the way for novel medical treatments, improved risk prediction, and refined strategies.

## 4. Clinical Evidence from Cohorts

Across large cohort studies, aneurysm-level characteristics predominantly determine rupture risk. Aneurysm size demonstrates a nonlinear association with rupture hazard, typically increasing beyond 7 mm, as confirmed in the UCAS-Japan study [20,47,48]. Location is also critical—aneurysms of the anterior communicating artery (Acom), posterior communicating artery (PcoA), and posterior circulation aneurysms exhibit higher rupture rates than middle cerebral artery or internal carotid artery lesions. Irregular morphology, such as lobulation or the presence of a daughter sac, further amplifies rupture risk, reflecting focal hemodynamic stress and wall fragility. Pooled risk models, such as PHASES, integrate these variables to generate a pragmatic 5-year absolute rupture risk estimate for clinical counseling [21]. Documented interval growth serves as a short-term risk accelerator, with multicenter analyses reporting approximately 4% 1-year rupture rate after aneurysm growth, particularly in anatomically unfavorable sites [49]. Even small aneurysms are not risk-free, as demonstrated in the Small Unruptured Intracranial Aneurysm Verification study [50].

### 4.1. Patient-Level Factors

Smoking, hypertension, and prior SAH consistently increase the risk; familial predisposition and autosomal dominant polycystic kidney disease (ADPKD) increase baseline aneurysm prevalence and may shift the risk thresholds [46,51,52,53,54,55,56]. These factors should modulate the thresholds for treatment and surveillance.

### 4.2. Risk Scores and Clinical Judgment

For rupture risk, the PHASES offers an absolute 5-year estimate to anchor shared decision-making. For surveillance planning, ELAPSS predicts 3- and 5-year aneurysm growth and can guide follow-up intervals; once growth is observed, management should be time-sensitive. UCAS-Japan is best treated as foundational natural history evidence rather than as a standalone score, and the Unruptured Intracranial Aneurysm Treatment Score (UIATS) helps formalize treatment inclination when clinical factors are borderline by contrasting points favoring treatment versus observation.

PHASES provides a practical 5-year rupture estimate for UIA counseling [52]. Clinical judgment that integrates size, site, shape, growth, and patient factors typically outperforms any single tool [52]. Nina and colleagues recommend using the UIATS to structure treatment decisions for unruptured IAs (UIA); in this framework, a ≥+3-point difference in favor of treatment indicates candidacy for intervention, whereas ≤−3 points supports observation, and −2 to +2 is inconclusive [57]. UIATS sums factors across three domains: patient (age, life expectancy/comorbidity, prior SAH, family history, smoking, hypertension, and patient preference), aneurysm (size, location, morphology/irregularity, documented growth, multiplicity, and symptoms), and treatment (estimated procedural risk, durability of microsurgical vs. endovascular therapy, and technical complexity/institutional expertise).

## 5. Clinical Imaging & Quantitative Biomarkers

### 5.1. Vessel-Wall MRI (VWI)

High-resolution VWI (vessel-wall imaging) often shows circumferential thick enhancement in unstable UIA and correlates with histological inflammation and neovascularization [44,45,58,59,60]. Recent validation studies have demonstrated that quantitative measures of wall enhancement, such as CR₍stalk₎ or enhancement index, derived from HR-VWI, provide independent predictive value for rupture risk when incorporated alongside morphological and clinical factors [61,62]. However, size confounding is substantial, and flow-related pseudo-enhancement can mimic wall uptake, warranting a careful protocol and interpretation [60,63]. At present, VWI is best used as a complementary marker alongside morphology and growth, pending size-adjusted prospective outcome data. Moreover, emerging evidence suggests that systemic biomarkers, such as apolipoprotein A1 levels, may correlate inversely with wall enhancement on VWI, indicating a potential link between atherosclerotic processes and aneurysm wall instability [64]. Conversely, wall calcification on CT angiography (CTA) has been reported as a protective feature; calcified aneurysms tend to carry a lower rupture risk, although calcification may increase procedural complexity if treatment is performed. Therefore, calcification is considered an informative stability marker that can nudge decisions toward surveillance when other factors are borderline [65].

Beyond qualitative wall enhancement, several quantitative VWI metrics (e.g., aneurysm-to-pituitary stalk contrast ratio and extent of the enhancing wall) have been associated with instability in single-center studies. However, size confounding, flow-related pseudo-enhancement, and heterogeneous acquisition/segmentation currently limit generalizability. Standardized protocols, size-adjusted analyses, and prospective outcome-linked studies are needed to define the incremental value of VWI beyond the established clinical determinants (size/site/shape/growth). Notably, a recent multivariable model incorporating HR-VWI features together with parent artery wall enhancement achieved an area under the curve of 0.814 for rupture prediction, outperforming PHASES and ELAPSS scores in a validation cohort [66]. Practically, VWI serves as the most useful complementary marker for stratifying follow-up intensity and guiding interventions in borderline cases [67,68].

### 5.2. Shape and Hemodynamics (Clinically Usable Markers)

Adverse geometry, that is, high aspect ratio (AR) and size ratio (SR), was associated with rupture beyond size in multiple datasets [59,63,69]. Hemodynamic surrogates such as low wall shear stress (WSS), high oscillatory shear index (OSI), and increased low-shear area co-localize with fragile regions and growth foci [16,17,63,70,71]. Practically, AR ≥ 1.6 and irregular morphology are pragmatic “red flags” that should be interpreted with site/patient context. The meta-analysis by Zhou et al. evaluated mean wall shear stress and found that only two reports suggested high wall shear stress as a contributing factor to rupture. However, comparisons between patient-specific and generalized hemodynamic parameters revealed that patient-specific wall shear stress values were higher in ruptured aneurysms [72]. Nuclear factor kappa B (NF-κB), a pro-inflammatory transcription factor, acts upstream of endothelial gene expression in response to shear stress. Moreover, because hypertension is a crucial determinant in aneurysm formation in other vascular models, such as aortic aneurysms, these findings collectively indicate that elevated wall shear stress is a key factor in aneurysm initiation and progression [73,74,75,76].

### 5.3. Wall Motion of IAs

Recently, the abnormal wall motion (“irregular pulsation”) of intracranial aneurysms has attracted increasing attention. Dynamic four-dimensional computed tomography angiography (4D-CTA) enables visualization of subtle wall pulsations throughout the cardiac cycle, providing insight into wall instability. Prospective analyses, including that by Zhou et al., demonstrated that the presence of irregular wall pulsation on 4D-CTA is an independent predictor of rupture, even after adjustment for aneurysm size and site [77,78,79]. When such irregular pulsation newly appears or worsens during follow-up, it is considered a short-term red flag for rupture risk—particularly in anterior or posterior communicating artery lesions or in aneurysms with new lobulation. Integrating 4D-CTA findings with aneurysm growth and vessel-wall-enhancement imaging offers a pragmatic, multimodal approach to evaluating near-term rupture risk.

### 5.4. Radiomics and AI

Emerging radiomics and machine learning models integrate morphology, hemodynamic surrogates, and VWI features. Early studies suggested an add-on discriminatory value compared with size alone, but reproducibility across scanners, centers, and pipelines remains a barrier. External validation and calibration against prospective ruptures are prerequisites before routine adoption; meanwhile, these tools can be used exploratorily to prioritize follow-up or inform case discussions at multidisciplinary conferences [80].

## 6. Translational Clinical Implications

### 6.1. Risk Stratification Pathway (At the Hospital Level)

Start with PHASES for a 5-year anchor and then up-adjust risk in the presence of growth, high-risk site, irregularity, and/or VWI/geometry. In high-risk constellations, treatment is discussed even at modest sizes [18,19,49,53,56,59,60,69,81,82].

### 6.2. Surveillance Design

Use ELAPSS to tailor imaging intervals with short-interval follow-up after growth or when morphology is at high risk [47,48,63]. Clearly, the post-growth rupture risk is front-loaded over the next six to twelve months [49].

### 6.3. Therapeutic Directions

Mechanism-informed prevention strategies have also emerged. Observational data suggest that statins may reduce rupture risk via their anti-inflammatory effects, although randomized evidence is lacking [41,42,43]. Experimental targets—EP2/NF-κB signaling, C5a–C5aR1, angiogenesis, and neutrophil-mediated MMP-9—offer avenues for trials integrating wall imaging as a pharmacodynamic biomarker [6,9,12,21,36,37,38,44,45,49,58].

## 7. Clinical Therapy

The treatment of IAs can be broadly categorized into microsurgical (direct) approaches and endovascular interventions. 

The history of microsurgical management dates back to 1748 [77], when the proximal ligation of a cerebral aneurysm was first reported, and the modern clipping technique was introduced in 1911 [78]. Subsequent technological advances have significantly improved intraoperative visualization. Intraoperative fluorescence angiography was introduced in 2003 to confirm vascular occlusion in real time, and in 2006, the first endoscopic-assisted clipping surgery was reported worldwide [79].

Endovascular therapy has evolved in parallel. The first detachable balloon embolization was described in 1978, followed by the advent of coil embolization in 1990, which revolutionized minimally invasive aneurysm management [83,84]. The development of stent-assisted coiling (SAC) in 1997 expanded indications to complex and wide-neck aneurysms, enabling durable packing and parent vessel protection [85]. A major breakthrough came in 2008 with the introduction of the flow diverter (FD), specifically, the Pipeline Embolization Device (PED), which became the cornerstone for treating large, giant, and wide-neck sidewall aneurysms [86].

More recently, the Woven EndoBridge (WEB) (Terumo neuro, Irvine, CA, USA) device, first reported in 2011, has provided a novel intrasaccular flow disruption strategy, particularly advantageous for bifurcation aneurysms without the need for long-term dual antiplatelet therapy [87].

Together, these advances illustrate the progressive transition from invasive microsurgical clipping to individualized, anatomy-guided, and minimally invasive endovascular therapy. The management of IAs has evolved significantly over the past two decades, with treatment selection increasingly guided by aneurysm morphology, rupture risk, and patient-specific factors. Microsurgical clipping remains a durable and definitive option, particularly for younger patients and complex bifurcation aneurysms, offering long-term occlusion rates exceeding 90%. In contrast, multiple large-scale meta-analyses have consistently shown that endovascular coiling carries a significantly higher risk of aneurysm recurrence and retreatment compared to clipping, despite its lower procedural morbidity and mortality rates [88].

Endovascular coiling, introduced in the 1990s, provides a minimally invasive alternative with shorter hospital stays and lower immediate procedural risk. However, recurrence and retreatment rates remain higher, particularly in wide-neck or large aneurysms. For such cases, SAC or balloon remodeling is often employed to achieve stable packing density. Randomized trials, such as the STAT, have not shown clear superiority of SAC over conventional coiling in terms of long-term occlusion, and the requirement for dual antiplatelet therapy remains a limitation in acute or ruptured cases [89,90].

The introduction of FD stents, most notably the PED, has transformed the treatment landscape for large or giant sidewall aneurysms. In the PUFS and PREMIER studies, PED achieved complete occlusion rates of 76–85% at 1 year and low retreatment rates (approximately 5%), with acceptable periprocedural complication rates (<6%) [91,92,93,94]. Recent meta-analyses have confirmed these outcomes and expanded indications to distal and bifurcation aneurysms using newer low-porosity or surface-modified devices [95].

More recently, intrasaccular flow disruption devices, such as the WEB (Terumo, Irvine, CA, USA), have emerged as effective options for bifurcation aneurysms, offering favorable occlusion rates with reduced need for long-term antiplatelet therapy. The WEBCAST and WEB-IT trials, including 5-year follow-up data, demonstrated adequate occlusion in approximately 80% of aneurysms and low retreatment rates (<10%), confirming durable long-term efficacy and safety [96,97,98].

Collectively, these advances reflect a paradigm shift toward minimally invasive, anatomy-tailored interventions. Optimal management now requires a multidisciplinary approach that integrates morphology, VWI, hemodynamic parameters, and patient comorbidities to select the most appropriate individualized therapy (The summary of clinical therapy is Table 1).

## 8. Gene-Editing Therapy

### 8.1. Preclinical Proof-of-Concept (SOX17-CRISPRa)

Recent preclinical research has proposed endothelial stabilization as a disease-modifying strategy. CRISPR activation (CRISPRa)-mediated upregulation of SOX17 enhanced protective endothelial programs and reduced inflammatory and pro-degenerative signaling in IA models, establishing proof-of-concept for gene-editing–guided aneurysm wall stabilization (preclinical stage as of 2025) [99].

### 8.2. Human Genetic and Functional Rationale for SOX17 Targeting

Genome-wide association studies have consistently implicated SOX17 among IA susceptibility loci, supporting a causal link between endothelial regulatory networks and aneurysm biology. Mechanistic investigation further demonstrated that SOX17 enhancer variants disrupt transcription-factor binding and downstream endothelial function, thereby strengthening the translational rationale for SOX17-directed editing or activation [100].

Targeted delivery remains the principal challenge for clinical translation. Achieving selective delivery of gene-editing tools to the aneurysm wall, specifically to endothelial cells, vascular smooth muscle cells (VSMCs), and fibroblast-like cells, is essential for future application. Recent studies have identified engineered adeno-associated virus capsid variants with enhanced tropism for VSMCs [101] suggesting that cell type–specific delivery is becoming increasingly feasible. Nevertheless, optimizing delivery efficiency and vascular selectivity, particularly for intracranial arteries, as well as establishing safe and effective local (intra-arterial) administration methods, remain key developmental challenges.

Systematic reviews to date conclude that gene therapy for IAs remains in its early evidence-generating phase and requires further preclinical validation before clinical translation [102].

## 9. Limitation and Future Direction

Heterogeneity of populations, imaging protocols, and analytical methods limits direct comparability across studies. Size-adjusted prospective designs are needed to quantify the incremental value of VWI, 4D-CTA pulsation, and geometry/CFD beyond established clinical determinants. Cross-center standardization and external validation of quantitative workflows (including radiomics/AI) are priorities. Translationally, targets implicated by basic research—EP2/NF-κB, C5a–C5aR1, angiogenesis, and neutrophil-mediated MMP-9—merit evaluation in early-phase trials that incorporate wall imaging as a pharmacodynamic readout. Such integrative designs may refine thresholds for interventions and enable mechanism-based prevention.

## 10. Conclusions

This article summarizes recent insights into the pathophysiology of IA, with a focus on rupture mechanisms and clinical features. Ruptures arise from a transition to a neutrophil-rich, hypoxia-driven, angiogenic inflammatory microenvironment superimposed on adverse hemodynamics and susceptible genetics. Integrating clinical determinants (size, site, shape, and growth) with VWI and geometry and hemodynamics can sharpen risk stratification, whereas mechanism-based therapies and standardized imaging may enable targeted prevention in the future.

## Figures and Tables

**Table 1:** Comparison of clinical therapy.

Category	Milestone/Device (Year)	Key Features and Advances	Advantages	Limitations/Notes	Key References
Microsurgical approaches	Proximal ligation (1748)	First reported ligation of a cerebral aneurysm.	Historical origin of direct surgical repair.	Non-selective, high morbidity.	[86]
	Clipping technique (1911)	Introduction of aneurysm neck clipping; foundation of modern surgery.	Durable complete occlusion (>90%).	Invasive; technically demanding.	[87]
	Intraoperative fluorescence angiography (2003)	Real-time confirmation of vascular patency and occlusion.	Improved safety and accuracy.	Limited to open surgery.	[88]
	Endoscopic-assisted clipping (2006)	First reported use of endoscope for visualization in clipping surgery.	Better visualization of deep/bifurcation lesions.	Requires microsurgical expertise.	[88]
Endovascular interventions	Detachable balloon embolization (1978)	First endovascular occlusion method.	Pioneering minimally invasive concept.	Technically limited; risk of migration.	[89]
	Coil embolization (1990)	Introduction of detachable coils (Guglielmi).	Minimally invasive; shorter hospital stay; lower perioperative risk.	Higher recurrence/recanalization rates.	[90]
	Stent-assisted coiling (SAC, 1997)	Use of stents to support coil packing in wide-neck aneurysms.	Expanded indications to complex aneurysms; stable packing.	Dual antiplatelet therapy required; not ideal for ruptured cases.	[89,90]
	Flow diverter (FD, 2008, Pipeline Embolization Device [PED])	Paradigm shift to parent-vessel reconstruction.	Complete occlusion 76–85% at 1 year (PUFS, PREMIER); low retreatment (~5%).	Procedure-related risk <6%; limited initially to large sidewall aneurysms.	[91,92,93,94,95]
	Intrasaccular flow disruption (WEB, 2011)	Woven EndoBridge device for bifurcation aneurysms.	Effective occlusion (~80% at 5 years); no long-term dual antiplatelet therapy needed.	Limited to specific morphologies; ongoing device refinement.	[96,97,98]
Current direction	-	Integration of morphology, VWI, and hemodynamics for individualized, anatomy-tailored therapy.	Multidisciplinary optimization of treatment choice.	-	-

## Data Availability

Not applicable. No new data were created or analyzed in this study.
